# Mononuclear cell transcriptome changes associated with dimethyl fumarate in MS

**DOI:** 10.1212/NXI.0000000000000470

**Published:** 2018-06-12

**Authors:** Arie R. Gafson, Kicheol Kim, Maria T. Cencioni, Wim van Hecke, Richard Nicholas, Sergio E. Baranzini, Paul M. Matthews

**Affiliations:** From the Division of Brain Sciences (A.R.G., M.T.C., R.N.), Department of Medicine, Imperial College London; the Department of Neurology (K.K.), Weill Institute for Neurosciences, University of California, San Francisco; icometrix (W.v.H.), Begaultlaan, Leuven, Belgium; the Department of Neurology (S.E.B.), Weill Institute for Neurosciences, Institute for Human Genetics and Graduate Program in Bioinformatics, University of California, San Francisco; and Division of Brain Sciences (P.M.M.), Department of Medicine, the Centre for Neurotechnology and the UK Dementia Research Institute, Imperial College London.

## Abstract

**Objective:**

To identify short-term changes in gene expression in peripheral blood mononuclear cells (PBMCs) associated with treatment response to dimethyl fumarate (DMF, Tecfidera) in patients with relapsing-remitting MS (RRMS).

**Methods:**

Blood samples were collected from 24 patients with RRMS (median Expanded Disability Status Scale score, 2.0; range 1–7) at baseline, 6 weeks, and 15 months after the initiation of treatment with DMF (BG-12; Tecfidera). Seven healthy controls were also recruited, and blood samples were collected over the same time intervals. PBMCs were extracted from blood samples and sequenced using next-generation RNA sequencing. Treatment responders were defined using the composite outcome measure “no evidence of disease activity” (NEDA-4). Time-course and cross-sectional differential expression analyses were performed to identify transcriptomic markers of treatment response.

**Results:**

Treatment responders (NEDA-4 positive, 8/24) over the 15-month period had 478 differentially expressed genes (DEGs) 6 weeks after the start of treatment. These were enriched for nuclear factor (erythroid-derived 2)-like 2 (*Nrf2*) and inhibition of nuclear factor κB (NFκB) pathway transcripts. For patients who showed signs of disease activity, there were no DEGs at 6 weeks relative to their (untreated) baseline. Contrasting transcriptomes expressed at 6 weeks with those at 15 months of treatment, 0 and 1,264 DEGs were found in the responder and nonresponder groups, respectively. Transcripts in the nonresponder group (NEDA-4 negative, 18/24) were enriched for T-cell signaling genes.

**Conclusion:**

Short-term PBMC transcriptome changes reflecting activation of the Nrf2 and inhibition of NFκB pathways distinguish patients who subsequently show a medium-term treatment response with DMF. Relative stabilization of gene expression patterns may accompany treatment-associated suppression of disease activity.

Relapsing-remitting MS (RRMS) is an autoimmune disease affecting the CNS. Many disease-modifying treatments (DMTs) are available, but none have efficacy in all patients, all are expensive, and all are associated with possible adverse events.^1^ Stratifying patients to the best tolerated and most efficacious treatment either before or soon after commencing treatment would enhance relative benefits and reduce harm.^2,3^

Currently, the most common approach to ensuring that patients are receiving efficacious medication involves vigilant disease monitoring using clinical measures and serial MRI. The most recent composite “no evidence of disease activity” (NEDA-4) outcome measure combines 4 indices of disease activity: relapses, Expanded Disability Status Scale (EDSS) progression, new MRI activity (Gd+ lesions or new/newly enlarging T2 lesions), and relative brain volume loss.^4,5^ While sensitive to disease activity, a limitation of this approach is the time of observation required to meaningfully assess changes in these component measures. There is a need for shorter term predictive markers of clinical outcomes.

Dimethyl fumarate (DMF) (BG-12; Tecfidera) is a first-line therapy of moderate efficacy approved for use in RRMS. However, a recent post hoc analysis revealed that only 26% of patients in the original DEFINE/CONFIRM trials were without clinical evidence of disease activity or new or enlarging T2-hyperintense lesions on MRI at 2 years.^6^ This suggests that there may be responder and nonresponder populations. However, currently, there is no early response stratification marker to distinguish them. The hypothesized mechanism of action involving modulation of nuclear factor (erythroid-derived 2)-like 2 (*Nrf2*)^7,8^ and nuclear factor κB (NFκB)^9^ in immune cells suggested that pharmacodynamic effects on the transcriptome of peripheral blood mononuclear cells (PBMCs) could be used to predict treatment response.^10^ To test this, we used next-generation RNA sequencing to identify short-term changes in gene expression at 6 weeks after treatment initiation that are associated with medium-term treatment response defined by the composite outcome measure NEDA-4 at 15 months after treatment.

## Methods

### Standard protocol approvals, registrations, and patient consents

Our research study was reviewed and approved by the NHS Research Ethics Committee of London Camden and Islington (14/LO/1896). All patients provided written informed consent.

The study cohort included 24 patients with RRMS (median EDSS score, 2; range 1–7) recruited from the Imperial College Healthcare NHS Trust. Inclusion criteria were diagnosis of RRMS by McDonald criteria,^11^ age between 18 and 65 years, intent to commence DMF, and otherwise treatment-free (other DMTs or steroids) for at least 3 months before sample collection. Exclusion criteria were known or suspected intolerance or contraindication to MRI. Seven age- and sex-matched healthy volunteers who were not receiving any prescribed or over the counter medicines were recruited by local advertising.

Patients and healthy volunteers attended for 3 study visits. For patients, these were at baseline, before the onset of treatment, 6 weeks after the initiation of treatment with DMF, and after 15 months of DMF treatment. In the volunteer cohort, there were also 3 study visits over the same time intervals, but no drug was taken. The EDSS was conducted by a single, trained physician (A.R.G.).

### Sample collection

Nonfasting venous blood samples were collected at each study visit in EDTA tubes. PBMCs were extracted from fresh whole blood within 1 hour of sample collection using a Ficoll gradient (Histopaque-1077; Sigma Life Science, St. Louis, MO). Buffy coat containing PBMCs was aspirated using sterile Pasteur pipettes and washed with sterile phosphate-buffered saline (PBS). Two wash cycles were performed, followed by resuspension of cell pellet in 10 mL PBS. Cells were then counted using Trypan blue, and aliquots of 5–10 million cells were taken for RNA extraction. RNA extraction was performed on fresh pellet directly after PBMC extraction using the Qiagen RNeasy kit as per the manufacturer's guidelines (Qiagen, Hilden, Germany).

### RNA-Seq protocol

RNA was maintained at −80°C before sequencing. The quality of the RNA prepared was confirmed, and all samples were sequenced at the same time. Quality was assessed as the RNA concentration measured using a Nanodrop and Qubit 2.0 Fluorometer (Life Technologies, Carlsbad, CA), and RNA integrity was tested using TapeStation (Agilent Technologies, Palo Alto, CA). All samples showed a 260-nm/280-nm fluorometer intensity ratio of >2 and an RNA integrity number of >9.

Library preparation was performed using the Illumina NEBNext Ultra RNA Library Preparation Kit following the manufacturer's recommendations (NEB, Ipswich, MA). Messenger RNA was enriched with Oligo d(T) beads and then denatured for 15 minutes at 94°C. Complementary DNA (cDNA) was then synthesized, end paired, and adenylated at 3′ ends. Then, a universal adapter was ligated to cDNA fragments along with the index sequence, and the library was further enriched with limited cycle PCR. The size of the resulting sequencing libraries was measured using Agilent TapeStation and quantified using Qubit 2.0 Fluorometer and quantitative PCR (Applied Biosystems, Carlsbad, CA). RNA sequencing was performed on an Illumina HiSeq platform (San Diego, CA) (2 × 150 base pairs; paired-end configuration). The quality of the raw sequence data (Fastq files) was assessed by Phred scoring. The Phred Q score of 30 was >96% for all samples.

Raw sequences were aligned to the human reference genome GRCh38.p10 using Dynamic Read Analysis for Genomics software. Gene hit counts were calculated from the output BAM files using HTSeq-count, a python library that counts aligned reads overlapping exons for each gene.^12,13^ Only reads mapping unambiguously to a single gene are counted using the software, and reads possibly mapping to more than 1 gene are discarded.

### MRI scans

The patients with RRMS underwent MRI at the Imperial College Clinical Imaging Facility at 6 weeks and 15 months after the start of treatment (Siemens Verio 3T; 32-channel head coil; T1-and T2-weighted structural scans). Scans were analyzed using MSmetrix, a scanner-independent and clinically approved software developed by Icometrix to extract whole brain atrophy, lesion volume changes, and the number of new lesions between 2 timepoints.^14,15^ The longitudinal approach taken by MSmetrix incorporates both spatial and temporal information for accurate and consistent lesion segmentation based on Markov Random Field modeling and difference imaging across the 2 timepoints.^16^

### Clinical outcomes

Clinical outcomes of patients were assessed by a single, trained physician (A.R.G.) before the onset of treatment, 6 weeks after the initiation of treatment with DMF, and after 15 months of DMF treatment (A.R.G.). At each clinical visit, detailed patient histories were taken including data concerning any new clinical relapses over the study period, a full EDSS assessment, MS Functional Composite (MSFC) scoring,^17^ and 36-item Short-Form (SF-36)^18^ quality-of-life questionnaire.

The NEDA-4 outcome measure was used to designate patients as responders or nonresponders at 15 months. NEDA-4 was defined as no evidence of relapses, active MRI lesions (both new or enlarged T2 lesions), 6-month confirmed disability progression (CDP) (defined as an increase in the EDSS score of 1.5 points from a baseline score of 0, of 1.0 point from a baseline score of 1.0 or more, or 0.5 points from baseline greater 5.0), or a mean annualized rate of brain volume loss (AR-BVL) of more than 0.4%.^5^ Secondary outcome measures included MSFC Z-score comparisons at baseline and 15 months. The SF-36 was scored at baseline and 15 months as a Physical Summary Score (PCS) and a Mental Component Score (MCS).

### Statistical analyses

Treatment responders and nonresponders as classified by NEDA-4 criteria were studied using both time-course and cross-sectional analyses. Independent differential expression analyses were performed on count data derived from HT-Seq using DESeq2^19^ for the responder and nonresponder groups. DESeq2, which performs differential expression analysis by first performing a regularized logarithm transformation, followed by detection and correction of dispersion estimates that are too low through modeling using average expression strength over all samples, was used for this. An assumption made by this software is that genes of similar average expression have similar dispersion. Where counts are low or dispersion is high for a specific gene, DESeq2 shrinks the log-fold change (LFC) toward zero.^20^ The software provides an LFC value across conditions and an adjusted *p* value corrected for multiple comparisons using the Benjamini-Hochberg^20^ method.

Time-course differential expression contrasts were performed between baseline and 6-week samples and then between 6-week and 15-month samples, using a paired approach that controlled for intraindividual variations. Cross-sectional contrasts between patients and controls were performed, controlling for age as a covariate. Adjusted *p* value for significance (Padj) was set at Padj < 0.05. Fold change cutoffs were thresholded at log2-fold change of ±0.3.

Downstream pathway analysis was performed using Ingenuity Pathway Analysis (IPA) software (Qiagen). Genes of interest imputed into IPA had Padj < 0.05 and log2-fold change of ±0.3. Enrichment analysis was performed using g:Profiler isolated to KEGG pathways (*p* < 0.0005).

MSFC Z-score and SF-36 comparisons at baseline and 15 months were calculated using a paired Student *t* test. Permutation analysis was performed using DESeq2 (100-fold) with random selection of patients with RRMS (n = 8). The significance of differences in distributions derived from leave-one-out cross-validation (LOOCV) was determined using a Student *t* test (*p* < 0.05).

### Data availability

Anonymized data will be shared by request from any qualified investigator.

## Results

Baseline demographics and clinical information of patients and approximately age- and sex-matched healthy controls are shown in table 1. Eight patients had been on a previous DMT (although none received drug in the 3 months preceding sample collection), and 16 were treatment naive. Two patients had thalassemia trait, 2 had psoriasis, and 2 had autoimmune thyroid disorders. Table e-1, links.lww.com/NXI/A50 lists concurrent medications taken by patients with RRMS.

**Table 1:**
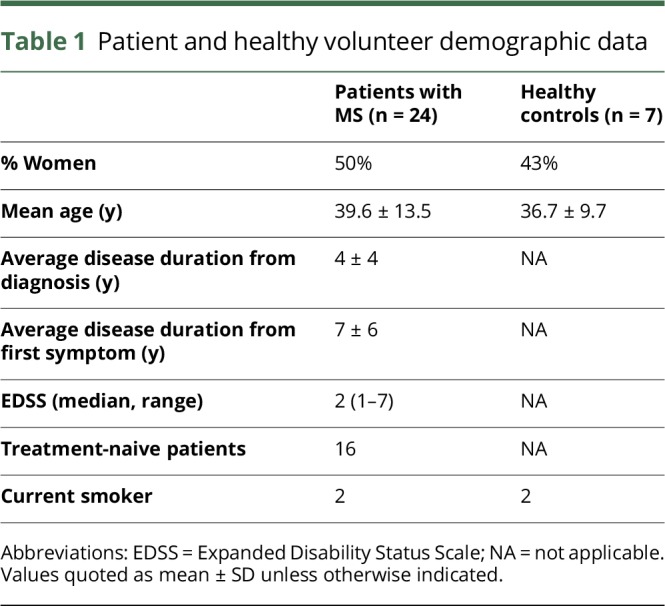
Patient and healthy volunteer demographic data

NEDA-4 was achieved by 8/24 patients (33%) over the 15-month period after initiating treatment with DMF. An AR-BVL greater than −0.4% (range, −0.44% to −2.19%) was found for 12 patients (50%). Enlarging or new lesions occurred in 9 patients (38% and 4 of these had an AR-BVL <−0.4%). Three patients experienced relapses, and 6-month CDP occurred in 4 patients (2/4 of whom also experienced relapses).

The median change in the MSFC score from baseline to 15 months for the whole cohort was +0.21 (range, −0.27 to 1.33) (*p* < 0.005). The median change in the SF-36 PCS was +4.4 (range, −39.4 to 51.25), and the median change in the MCS was +2.9 (range, −24.9 to 39.0), but these changes were not statistically different (*p* = 0.24 and *p* = 0.1, respectively).

### Short-term pharmacodynamic effects of DMF

We first tested for differentially expressed gene (DEG) between the healthy controls and all the patients with MS before the start of DMF, at baseline. Five hundred twenty-two genes were differentially expressed (DE) between patients and controls (Padj < 0.05). Of these, 254 were downregulated in patients and 268 were upregulated. There was enrichment of KEGG pathways “B-cell activation involved in the immune response” and “TNF signaling pathway” (*p* < 0.001).

**Figure 1:**
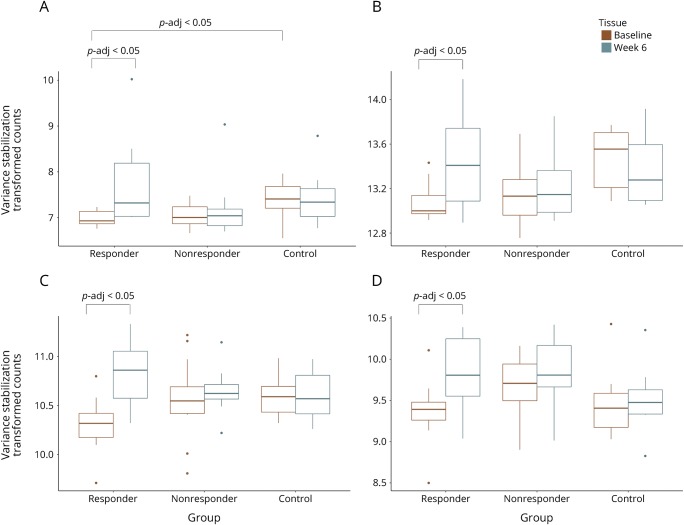
(A–D) Nuclear factor (erythroid-derived 2)-like 2–related transcripts are increased 6 weeks after treatment in responders but not in nonresponders or healthy controls Boxplots represent variance-stabilized transformed counts for transcripts (A) FOSL1, (B) ATF4, (C) MAFG, and (D) MGST1 at baseline and 6 weeks in responders, nonresponders, and healthy controls. ATF4 = activating transcription factor 4; FOSL1 = fos-related antigen 1; MAFG = transcription factor MafG; MGST1 = microsomal glutathione S-transferase 1.

**Figure 2:**
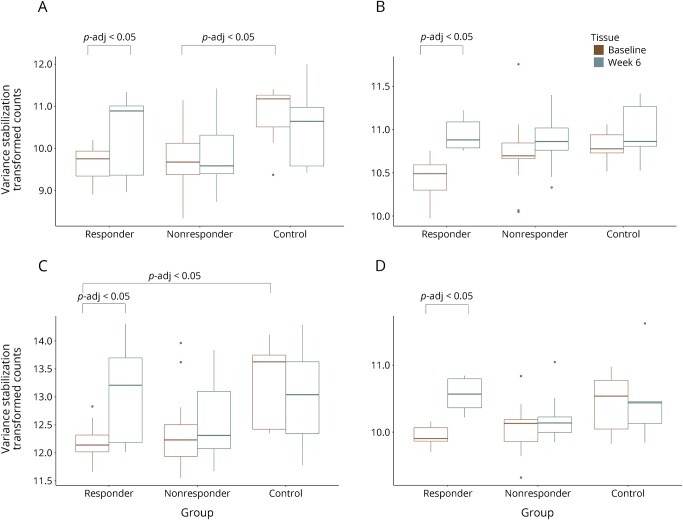
(A–D) NFκB-related transcripts are increased 6 weeks after treatment in responders but not in nonresponders or healthy controls Boxplots represent variance-stabilized transformed counts for transcripts (A) CD83, (B) ICAM1, (C) NFκBIA, and (D) NFκBIE at baseline and 6 weeks in responders, nonresponders, and healthy controls. CD83 = cluster of differentiation 83; ICAM1 = intercellular adhesion molecule 1; NFκB = nuclear factor κB; NFκBIA = nuclear factor of kappa light polypeptide gene enhancer in B-cells inhibitor, alpha; NFκBIE = nuclear factor of kappa light polypeptide gene enhancer in B-cells inhibitor, epsilon.

We assessed the pharmacodynamic effects of DMF in patients, independently testing for those in the clinical responder and nonresponder groups. In the responder group, there were 478 DEGs 6 weeks after the start of treatment with DMF relative to baseline (padj < 0.05). These differences showed enrichment of transcripts related to the Nrf2 pathway (*p* < 0.0005) (figure 1) and increased expression of those associated with inhibiting NFκB responses (overlap *p* < 0.0005) (figure 2). In the nonresponder group, no consistent DEGs were identified 6 weeks after the start of treatment relative to baseline (table 2).

**Table 2:**
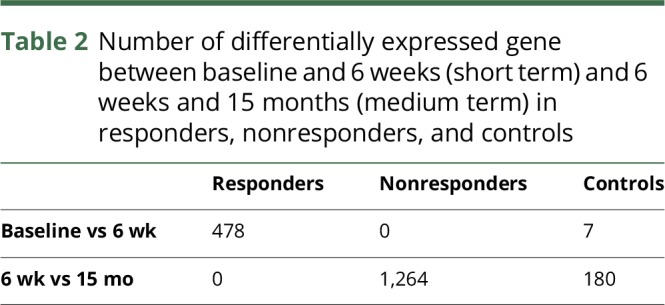
Number of differentially expressed gene between baseline and 6 weeks (short term) and 6 weeks and 15 months (medium term) in responders, nonresponders, and controls

We confirmed the significance of this difference in responder and nonresponder groups by testing for effects of outlier values using LOOCV. The median numbers of DEGs after treatment were 404 and 0 in the responder and nonresponder groups, respectively (*p* < 0.0005). We also assessed RNA-Seq data from untreated healthy controls (n = 7). Comparison from baseline to the end of a 6-week period without any intervention showed only 7 DEGs (padj < 0.05) (table 2).

### Treatment response is associated with a stable pattern of gene expression

Between 6 weeks and 15 months, 0 and 1,264 DEGs were detected in the responder and nonresponder groups, respectively (table 2). We further confirmed a difference between the 2 groups using a 100-fold permutation analysis in randomly selected combinations of 8 patients with RRMS. The median number of DEGs in this analysis was 702 (range, 31–3,230). In healthy controls (n = 7), who were not given any intervention and who were followed up over the same time period, there were 180 DEGs (table 2).

The large number of DEGs found between 6 weeks and 15 months in the nonresponder group prompted us to test for response heterogeneity within this group. We first tested for individual outliers. Based on PCA of the 16 nonresponders at 15 months, responses in 2 patients appeared as outliers and were therefore removed from further analysis (figure e-1, links.lww.com/NXI/A48). After the removal of these 2 outliers, 2 distinct nonresponder groups (arbitrarily called groups A and B) were identified in a subsequent round of PCA (figure e-2). We then independently assessed DEGs between 6 weeks and 15 months in these groups: 560 DEGs were found for group A and 648 for group B (117 [11%] of these DEGs overlapped between the 2 groups). We tested for the significance of these differences relative to the stable expression pattern in the responder group using LOOCV. The median numbers of DEGs with LOOCV were 270 and 497 for groups A and B, respectively, which both are different to the equivalent analysis for the responder group, in which the median number of DEGs was 0 (*p* = 0.03 and *p* = 0.004, respectively).

In group A, the most enriched canonical pathways were involved with Th1 and Th2 activation and T-cell receptor signaling (*p* < 0.0001). The DEGs showed enrichment for the KEGG pathway “T-cell receptor signaling” (*p* < 0.0001). A comparison of patients with healthy controls at 15 months also demonstrated enrichment of the T-cell receptor signaling pathway, suggesting that failure to respond well to treatment is associated with T-cell dysregulation. There were no enriched canonical or KEGG pathways in group B.

### DMF treatment is associated with short-term relative normalization of gene expression in responders

**Figure 3:**
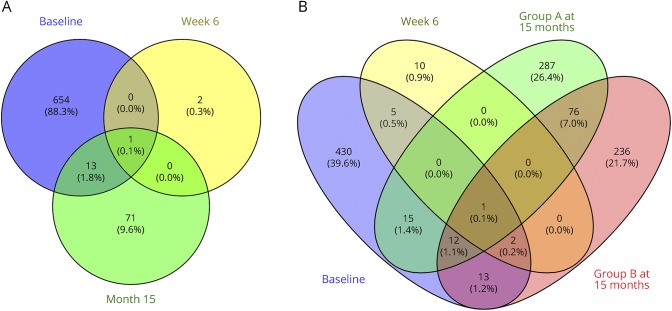
(A–B) Dimethyl fumarate treatment is associated with a relative normalization of gene expression in responders but not in nonresponders Venn diagrams represent the number of differentially expressed gene in responder (A) and nonresponder (B) groups compared with controls at baseline, 6 weeks, and 15 months.

After controlling for sex, 668 DEGs were found in samples from patients in the responder group relative to the healthy controls at baseline (padj < 0.05). However, 6 weeks after the start of treatment, only 3 genes were DE between these patients and the healthy controls (table 3). At 15 months, there were 85 DEGs between these patients and healthy controls, although only 14 genes (2%) overlapped with the DEGs found at baseline (figure 3A).

**Table 3:**
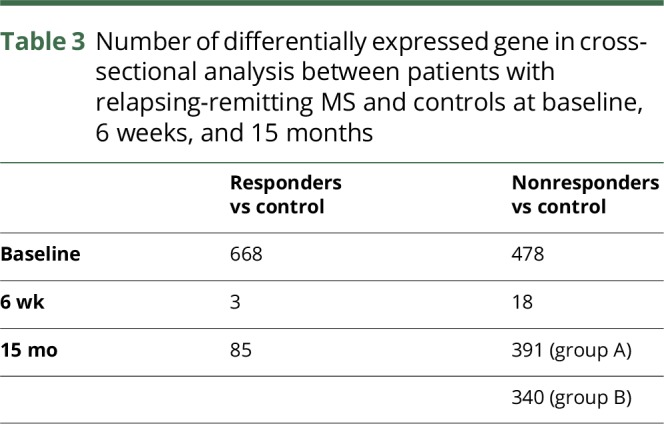
Number of differentially expressed gene in cross-sectional analysis between patients with relapsing-remitting MS and controls at baseline, 6 weeks, and 15 months

Four hundred seventy-eight DEGs were found in samples from patients in the nonresponder group relative to the healthy controls at baseline (padj < 0.05) (table 3). Ninety-eight (21%) overlapped with those identified from the responder group. At 6 weeks after the start of treatment, 18 genes were DE between the nonresponder patients and healthy controls, 8 of which (44%) also had been identified baseline (figure 3B). At 15 months, 391 DEGs were found in nonresponder group A and 340 in nonresponder group B (table 3).

## Discussion

There is currently no reliable early treatment response prediction marker for any RRMS DMT. Here, we explored the hypothesis that short-term, individual pharmacodynamic responses can distinguish patients who will respond to DMF. To do this, we tested whether gene expression changes at 6 weeks are associated with the medium-term clinical response to DMF. Using RNAseq, we observed that a robust short-term transcriptomic response to DMF in PBMCs was associated with activation of the *Nrf2* and inhibition of the *NFκB* pathways in treatment responders. In addition to a robust short-term pharmacodynamic response to DMF in treatment responders, we observed stabilization of gene expression between 6 weeks to 15 months. By contrast, no early transcriptional changes were observed after starting DMF in nonresponders. We also found greater expression of proinflammatory pathway genes in nonresponders than in healthy controls.

A number of previous studies also have investigated the pharmacodynamic effects of DMF on gene expression. These described modulation of genes related to antioxidant pathways,^21–24^ anti-inflammatory pathways,^25,26^ and NFκB^27,28^ that may be related to therapeutic effects. Our results confirmed changes in expression in all 3 of these pathways in the subset of patients in whom the drug suppresses apparent disease activity. We also demonstrated relative stabilization of gene expression over the medium term in treatment responders. By contrast, the nonresponders showed substantial numbers of DEGs over the same time period. In a subset of these nonresponders (group A), we found increased expression of immune activation pathways.

This study identified DEGs between patients with RRMS and healthy controls at baseline, before treatment. Although some previous studies also reported DEGs between these groups,^29,30^ our use of RNA-Seq allowed a wider range of discriminatory transcripts to be identified.^31^ However, PBMC expression differences between treatment-responsive RRMS patients and healthy controls did not persist after the initiation of treatment with DMF. We also provided data suggesting additional pharmacodynamic effects from stabilization of gene expression relative to treatment nonresponders. We speculate that the latter reflects enhanced immune homeostasis and suggest that the transcriptome differences relative to healthy controls and their dynamics may be markers of pharmacodynamic response in MS. Supportive evidence for the latter hypothesis comes from observations of dynamic transcriptome expression with respiratory syncytial virus infection of an otherwise healthy control monitored with repeated transcriptomics over 14 months.^32^

Overall, our treatment response frequency in this pragmatic, real-world study group was similar to that reported before in larger populations. We found that 33% of patients achieved NEDA-4 after 15 months of treatment. This is consistent with a recent post hoc analysis reporting on the medium-term NEDA outcomes of the phase III trials (DEFINE/CONFIRM) of DMF in RRMS.^6^ We also observed an improvement in the overall MSFC score at 15 months, as also reported from the initial DEFINE/CONFIRM studies.^33^ Although others have been able to show an improvement in QOL with DMF as measured by SF-36,^34^ we were unable to replicate this finding.

We acknowledge limitations of our work. The small sample size and using only 3 timepoints limit the confidence in our selection of transcriptomic response markers and estimation of their effect size. Further confirmatory work is needed. However, we attempted to reduce the effect of these factors by increasing the rigor of our statistical analyses (e.g., performing LOOCV and permutation tests). The use of a control group at matching timepoints also allowed us to compare our findings in the patients with those in a matched, healthy population while controlling for potential effects of time. We also minimized the effect of artefacts arising from “batch” effects by sequencing all samples at the same time and in the same sequencing facility.

To enhance the power to discriminate responders and the nonresponders, we used the NEDA-4 criteria, which rely on the assessment of the apparent rate of brain atrophy. In doing so over this short time frame (compared with that used for clinical decision making), we could not formally take into account the possibility of “pseudoatrophy.” We attempted to minimize the potential effect of this confound by “rebaselining” our patients after the initial 6 weeks on medication when greatest artefactual atrophy might take place.^35^ To the extent that we may have misclassified the true treatment response, this approach will underestimate the efficacy of DMF. However, the most likely effect of this will be to reduce the sensitivity to detection of transcriptomic outcomes discriminating treatment responders, rather than to generate false-positive signals. Finally, although we performed intragroup predictive testing with leave-one-out cross-validation, without an independent replication cohort, we were unable to test the predictive power of our findings formally.

We have provided evidence that DMF can alter PBMC transcriptome profiles of patients with MS even over the short term. The changes that we found support current hypotheses for mechanisms of action via activation of Nrf2 and suppression of NFκB pathways. In addition, we discovered evidence that a treatment response to DMF is associated with enhanced immune homeostasis that “normalizes” gene expression in the PBMC fraction. Validation and extension of these results may have implications for patient stratification for best use of DMF in other inflammatory conditions.^36,37^ Our work highlights the sensitivity of RNA-Seq transcriptomic pharmacodynamic measures of drug response. Although the RNA-Seq whole transcriptome assays of PBMCs now are relatively costly, RNA-Seq and related methods have the potential to be streamlined and provided at modest cost to become a future clinical laboratory assay if their value is demonstrated.

## Glossary

AR-BVL: annualized rate of brain volume loss
cDNA: complementary DNA
CDP: confirmed disability progression
DE: differentially expressed
DEG: differentially expressed gene
DMF: dimethyl fumarate
DMT: disease-modifying treatment
EDSS: Expanded Disability Status Scale
IPA: Ingenuity Pathway Analysis
LFC: log-fold change
LOOCV: leave-one-out cross-validation
MCS: Mental Component Score
MSFC: MS Functional Composite
NEDA: no evidence of disease activity
NFκB: nuclear factor κB
Nrf2: nuclear factor (erythroid-derived 2)-like 2
PBMC: peripheral blood mononuclear cell
PCS: Physical Summary Score
PBS: phosphate-buffered saline
RRMS: relapsing-remitting MS
SF-36: 36-item Short-Form
